# Sphingolipids and Brain Resident Macrophages in Neuroinflammation: An Emerging Aspect of Nervous System Pathology

**DOI:** 10.1155/2013/309302

**Published:** 2013-09-02

**Authors:** Emma Assi, Denise Cazzato, Clara De Palma, Cristiana Perrotta, Emilio Clementi, Davide Cervia

**Affiliations:** ^1^Department of Biomedical and Clinical Sciences, Unit of Clinical Pharmacology, CNR Institute of Neuroscience, “Luigi Sacco” University Hospital, University of Milan, 20157 Milan, Italy; ^2^E. Medea Scientific Institute, 23842 Bosisio Parini, Italy; ^3^Department for Innovation in Biological, Agro-Food and Forest Systems (DIBAF), University of Tuscia, 01100 Viterbo, Italy

## Abstract

Sphingolipid metabolism is deeply regulated along the differentiation and development of the central nervous system (CNS), and the expression of a peculiar spatially and temporarily regulated sphingolipid pattern is essential for the maintenance of the functional integrity of the nervous system. Microglia are resident macrophages of the CNS involved in general maintenance of neural environment. Modulations in microglia phenotypes may contribute to pathogenic forms of inflammation. Since defects in macrophage/microglia activity contribute to neurodegenerative diseases, it will be essential to systematically identify the components of the microglial cell response that contribute to disease progression. In such complex processes, the sphingolipid systems have recently emerged to play important roles, thus appearing as a key new player in CNS disorders. This review provides a rationale for harnessing the sphingolipid metabolic pathway as a potential target against neuroinflammation.

## 1. An Introduction to Sphingolipids 

During the last decades, sphingolipids have come to the fore for their involvement in signalling events that control a variety of cellular activities [1]. All sphingolipids are composed by a long-chain sphingoid base backbone (e.g., sphingosine), an amide-linked long-chain fatty acid and one of various polar head groups, that defines the various classes of sphingolipid subtypes, such as a hydroxyl group in ceramide, phosphorylcholine in sphingomyelin (SM), and carbohydrates in glycosphingolipids (GLSs). Sphingolipids are mainly present at the level of the membranes, of which they contribute to define physical and chemical properties. Some of the intermediate molecules of the sphingolipids metabolism (ceramide, sphingosine-1-phosphate (S1P), glucosylceramide and (GluCer), gangliosides) and their generating and modifying enzymes (neutral and acid sphingomyelinase (A-SMase), acid ceramidase, sphingosine kinase (SK), GluCer synthase, glycosyltransferases, *β*-galactosidase, and *β*-hexosaminidase) contribute to regulate cellular growth, differentiation, and apoptosis [2–6]. Sphingolipids have complex metabolic pathways that lead to the transformation of many sphingolipids in other sphingolipids and *vice versa* most often acting in concert to fine tune biological responses. In this respect, a relevant system is the so-called “sphingolipid rheostat,” that is, the relative amounts of ceramide, sphingosine, and S1P. Ceramide can be synthesised either *de novo* by the sequential action of serine palmitoyltransferase, (dihydro) ceramide synthase, and (dihydro) ceramide desaturase at the cytoplasmic leaflet of the membrane of the endoplasmic reticulum [7] or through the breakdown of SM by the activation of the catabolic enzymes sphingomyelinases. This occurs in the endolysosomal compartment [8], at the outer and inner leaflets of the plasma membrane [9–12] and through the newly discovered salvage pathway, consisting in the breakdown of complex sphingolipids into sphingosine and reacylation to produce ceramide [13]. Sphingosine can also be phosphorylated by SK1 and SK2 to produce S1P. Whereas ceramide is proapoptotic and inhibits autophagy, S1P enhances cell survival [14–16]. The “sphingolipid rheostat” has thus been proposed as one of the mechanisms that control the cell fate towards either apoptosis or survival. This regulatory action occurs within and contributes to the overall regulation of the inflammatory status as well as the vascular and cardiac functions [17].

A complex aspect of the “sphingolipid rheostat” is that ceramide can be converted to other sphingolipids with signalling properties. The level of intracellular ceramide is indeed controlled by its transformation in GluCer by the microsomal enzyme, UDP-glucose: ceramide d-glucosyltransferase also known as GluCer synthase, a transmembrane protein localised in the cis/medial Golgi. GluCer is involved in many cellular processes such as cell proliferation, differentiation, oncogenic transformation, and tumour metastasis, and more recently, it has been implicated in venous thrombosis and in the anticoagulant activity of protein C [18]. Moreover, GluCer contributes to the physical properties and physiological functions of membranes and serves as the precursor for hundreds of species of GLSs found in different mammalian cell types.

**Figure 1:**
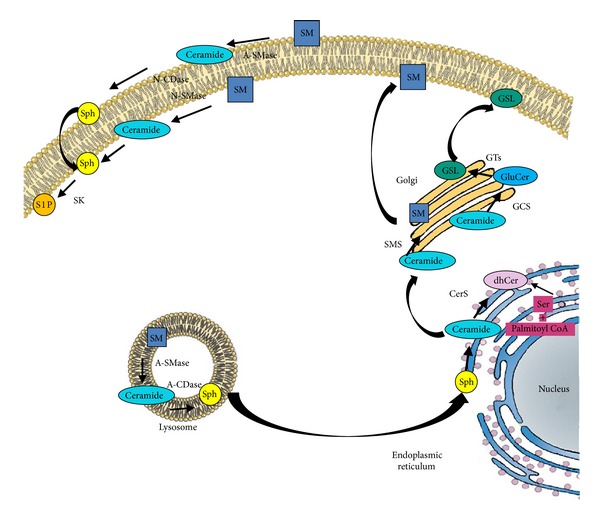
Schematic representation of main sphingolipid metabolic pathway. SM: sphingomyelin; Cer: ceramide; A-SMase: acid sphingomyelinase; N-SMase: neutral sphingomyelinase; A-CDase: acid ceramidase; N-CDase: neutral ceramidase; SMS: sphingomyelin synthase; Sph: sphingosine; S1P: sphingosine-1-phosphate; SK: sphingosine kinase; dhCer: dihydroceramide; CerS: ceramide synthase; GluCer: glucosylceramide; GCS: glucosylceramide synthase; GT: glycosyltransferase; GSL: ganglioside; Ser: serine.

Among them relevant are gangliosides GLSs containing sialic acid synthesised starting from GluCer, lactosylceramide, and galactosylceramide. Biosynthesis of these complex sphingolipids consists in the sequential addition of carbohydrate moieties to the existing acceptor glycolipid molecule and is catalysed by a series of specific glycosyltransferases localised in the Golgi apparatus [19]. The localisation of gangliosides in the outer leaflets of plasma membrane explains why they are involved in cell-cell recognition, adhesion, and signal transduction and are components of cell surface lipid rafts alongside proteins, SM, and cholesterol [5, 20–22]. A schematic representation of sphingolipid metabolic pathway is depicted in Figure 1.

Sphingolipid metabolism is deeply regulated along the differentiation and development of the central nervous system (CNS), and the expression of a peculiar spatially and temporarily regulated sphingolipid pattern is essential for the maintenance of the functional integrity of the nervous system [23–27].

## 2. Neuroinflammation and CNS Resident Macrophages 

There is a general agreement that neuroinflammation in nervous system disorders has active role in pathophysiology onset and progression, in conditions ranging from chronic pain and epilepsy to neurodegenerative diseases such as Alzheimer's disease (AD), Parkinson's disease (PD), lysosomal storage diseases, and amyotrophic lateral sclerosis and may even contribute to schizophrenia, depression, and other psychiatric disorders [28–30]. 

Many studies have focussed on the role of microglia in neuroinflammation and neurodegenerative diseases [31–35]. Indeed, in contrast to neurones and other glial cells, microglia are of haematopoietic origin and constitute the immune cells of the brain responding to pathogen infections and injuries. Microglial cells are specialised macrophages of the CNS distinct from other glial cells, such as astrocytes and oligodendrocytes, because of their origin, morphology, gene expression pattern, and functions [36–38]. They express macrophage-associated markers indicating a particularly close relationship with bone marrow-derived and thioglycollate-elicited peritoneal macrophages [38]. Indeed recent results in mice suggest that microglia originate from yolk sac macrophages that migrate into the CNS during early embryogenesis and are independent from cells that arise by definitive haematopoiesis in the bone marrow and from circulating cells [38]. 

Microglial cells are involved in phagocytosis and general maintenance of neural environment. Although these cells are quiescent under normal conditions, they are rapidly activated in response to pathological stimuli. On activation, resting microglia change their morphology immunophenotype and expression pattern of inflammatory mediators, leading to immune and inflammatory responses [35, 38–41]. In particular, activated microglial cells produce proinflammatory mediators, including cytokines, chemokines, reactive oxygen species (ROS), and nitric oxide (NO), which contribute to the clearance of pathogen infections. If prolonged or excessive, microglial cell activation may result in pathological forms of inflammation that contribute to the progression of neurodegenerative and neoplastic diseases [28, 38, 42]. Mechanisms that regulate the transition of microglia from the activated state associated with acute inflammation to phenotypes associated with tissue repair, and ultimately to phenotypes associated with normal CNS homeostasis, are poorly understood. 

Although it is still difficult to define conditions under which microglial cells are “good” or “bad,” strategies to counter the harmful effects of macrophage/microglial activation are studied widely to improve and enhance potential treatment strategies for disease conditions linked to neuroinflammation [28, 38, 43, 44]. In this respect, much current research has focussed on the signalling pathways that regulate inflammatory mediator production and subset macrophage development [38, 45]. Studies are still needed however to fully understand the role of these cells within the contexts of normal homeostasis and acute or chronic neuroinflammatory diseases [38, 45]. 

## 3. Sphingolipid Systems and Inflammation in CNS Diseases

The brain is one of the richest organs in lipid content; hence changes in the lipid levels may cause pathogenic processes. Publications from late 1980s and early 1990s suggested that decreased brain lipid levels and alterations in brain lipid metabolism are connected with AD. At present, there is a general consensus that, among CNS lipids, sphingolipid metabolism has a key neuropathological impact and sphingolipids have begun to be investigated in major CNS diseases [23, 24], including those related to inflammatory states [24, 25, 29]. In this respect, experimental evidence points to an important role for sphingolipids in inflammation [46, 47]. The contribution of sphingolipid metabolism to disease progression has received considerable attention because of increased levels of ceramide in the CNS under AD [29], X-adrenoleukodystrophy, and multiple sclerosis [48] and the demonstration of the ceramide role in induction of neural cell death [48, 49], proinflammatory gene expression [50, 51], and oxidative stress [48, 49, 51]. Overall, in CNS sphingolipids may be involved in regulating key intracellular events of cytokine signalling, in the production of the proinflammatory molecules eicosanoids [35], and in modulating cellular mechanisms as relevant as apoptosis, astroglial activation and astrogliosis, increase of T-cell migration, and activation of several receptor-mediated pathways [23, 25, 52, 53].

### 3.1. Alzheimer's Disease

There is evidence indicating that the increased brain-ceramide levels in AD lead not only to neuronal dysfunctions [54] but may also promote inflammatory processes [55]. The neuropathologic characteristics of AD are amyloid plaques (aggregates of amyloid-*β* peptides) and neurofibrillary tangles (formed by accumulation of hyperphosphorylated tau protein), which firstly affect the medial, temporal, and parietal lobes and part of the frontal cortex of the brain [56]. In primary oligodendrocyte cultures derived from neonatal rat brains A*β* peptide seems to activate SM hydrolysis causing ceramide accumulation [55]. Interestingly, ceramide influences the stabilisation of the enzyme *β*-secretase (BACE1), responsible for the cleavage of the amyloid precursor protein (APP) to form A*β* peptide [57] in human neuroglioma cells. The accumulation of A*β* peptide is responsible for the activation of microglia with subsequent release of large amounts of proinflammatory cytokines and ROS, contributing to the neuroinflammation and neurodegeneration [58]. Recent works on BV2 cells, a murine microglial cell line which is a suitable model for *in vitro* study of microglia, hypothesized that the inflammatory response of microglia in AD brains is mediated via S1P [59]. Recently, Tamboli and coworkers [60] have demonstrated a very interesting link between the storage of sphingolipids and the pathogenesis of AD, starting from the evidence that the presence of autophagosomes in dystrophic neurites is common to brains from patients affected by AD. The authors indicate that the accumulation of sphingolipids plays a dual role in autophagy; while promoting the induction of autophagy, sphingolipid may also impair the turnover of autophagic vesicles, leading to their accumulation and consequently to the accumulation of APP. 

### 3.2. Lysosomal Storage Disease Family

Other ceramide metabolites, that is, GLSs, are known to cause neurodegenerative and neuroinflammatory diseases, such as Gaucher, Fabry, mucopolysaccharidosis I and IIIA, and gangliosidosis [24, 29, 61]. Gangliosidosis is a GLS lysosomal storage disease in which the storage lipid is a GLS containing one or more sialic acid residue. It includes the GM2 storage disorders, Tay-Sachs and Sandhoff disease, and the GM1 storage disorders. Mouse models of the GM2 and GM1 gangliosidosis have been studied to determine whether there is a common neuroinflammatory component to these disorders. Of interest, Sandhoff disease mice treated with nonsteroidal anti-inflammatory drugs (indomethacin, aspirin, and ibuprofen) and antioxidants (L-ascorbic acid and-tocopherol acetate) lived significantly longer than untreated littermates and showed a slower rate of disease progression, thus suggesting that inflammation may play an important role in the pathogenesis of gangliosidosis [62]. In this respect, both GM2 and GM1 gangliosidosis mouse models exhibit progressive inflammatory reactions in the CNS which are characterized by altered blood brain barrier, apoptosis, and microglial activation with consequent release of proinflammatory cytokine [63]. It has been hypothesized that the microglial activation observed in these pathologies occurs via the Toll-like receptor 4 and that gangliosides may be involved in this process [39, 64]. 

### 3.3. Parkinson's and Huntington's Diseases

Targeting sphingolipid metabolism may also represent today an underexploited yet realistic opportunity to design novel therapeutic strategies for the intervention in PD. Indeed, it has been reported that the treatment with the monosialoganglioside GM1 restores at least partially neurochemical, pharmacological, histological, and behavioural parameters in different animal models of PD; it also reverses the dopaminergic deficits in nigrostriatal neurons of aged rats [27]. 

Several early studies suggested that altered sphingolipid metabolism is associated with Huntington's disease (HD) [27]. Noteworthy, a disrupted pattern of glycolipids (acidic and neutral lipids) and/or ganglioside levels was reported in both the forebrain of the R6/1 transgenic mice (a mouse model of HD) and caudate samples from human HD subjects [65]. However, although R6/1 transgenic mice have severe cerebellar GLS abnormalities that may account, in part, for their abnormal motor behaviour, the same abnormalities were not found in the cerebellum of human HD subjects [66]. The potential benefits of using gangliosides for treating the behavioural deficits associated with HD have also been described [67]. In particular, the administration of GM1 restores ganglioside levels in HD cells and promotes activation of the protein kinase Akt and phosphorylation of mutant huntingtin (htt) gene, leading to decreased mutant htt toxicity and increased survival of HD cells [68]. More recently, *in vivo* experiments demonstrated that intraventricular infusion of ganglioside GM1 induces phosphorylation of mutant htt at specific serine amino acid residues leading to attenuated htt toxicity and restores motor function in already symptomatic HD mice [69].

## 4. Sphingolipid Systems and Macrophages/Microglia Inflammatory Responses 

Implications for sphingolipids as signalling molecules for inflammatory responses come from various contexts [46, 47, 70]. Of interest, as comprehensively reviewed by Nixon [47], a significant body of research now indicates that sphingolipids are intimately involved in the inflammatory process and that these lipids, together with associated enzymes and receptors, can provide effective drug targets for the treatment of pathological inflammation. In some cell types sphingolipids can have specific effects that are integral to regulation of the inflammatory response. Sphingolipids themselves may, in certain circumstances, initiate parts of the inflammatory process [47]. However, at present, controversial reports have been presented on the beneficial versus detrimental role of sphingolipids and in particular ceramides in inflammation. Some studies have shown the proinflammatory role of ceramides in pulmonary oedema, airway inflammation, cystic fibrosis, and inflammatory bowel disease [71–76], whereas others have reported the anti-inflammatory effects of ceramides in macrophages, neutrophils, and corneal epithelial cells [74, 77–79]. 

As suggested before, modulation in macrophage/microglia phenotypes may contribute to pathogenic forms of inflammation and neurodegenerative diseases. Among sphingolipids, short-chain ceramides, commonly used to mimic the mechanisms of action of naturally occurring long-chain ceramides, have been demonstrated to have an anti-inflammatory effect in rodent macrophages. In particular, C2 to C8 short-chain ceramides reduce inflammation in cells stimulated by lipopolysaccharide (LPS), a bacterial polysaccharide commonly used as a proinflammatory stimulus. This anti-inflammatory effect was induced in part through the inhibition of cytokine, such as tumour necrosis factor (TNF-*α*), and chemokines, such as macrophage inflammatory protein 2, levels [78] and in part through the reduction of inducible NO synthase (iNOS) and cyclooxygenase-2 (COX-2) expression with consequent decrease of ROS level. These actions are accompanied by inhibition of several protein kinases, such as I*κ*B kinase, p38 mitogen-activated protein kinase (MAPK), and protein kinase C [80, 81]. Ceramide inhibits TNF-*α* secretion by regulating TNF*α*-converting enzyme activity [79] in mouse primary macrophages. Moreover, A-SMase knock-out mice showed an upregulation of serum TNF-*α* in response to LPS [82]. Noteworthy, C2 to C8 short-chain ceramides reduce inflammation in LPS-stimulated rodent microglia (BV2 cells and primary cultures), interfering with the binding of LPS to its cell surface receptors (i.e., TLR-4) and modulating intracellular pro-/anti-inflammatory signalling molecules [74]. In particular, C2 ceramide exerts its anti-inflammatory function also by reducing inflammation induced by TLR-2 or TLR-3 agonists and the phosphorylation of three types of kinases (MAPKs, Akt, and JAK) [74]. 

The possibility that long-chain ceramides mediate a proinflammatory effect in macrophages has been also demonstrated, thus suggesting that the function of ceramides differs depending on acyl chain length and cell types [6, 52, 74]. For instance, TNF-*α* activation of A-SMase results in the production of long-chain ceramides, C16–C24, with the subsequent activation of the proinflammatory transcription factor, nuclear factor-*κ*B (NF-*κ*B) [83]. NF-*κ*B is a family of ubiquitous transcription factors inducing more than 150 genes in different mammalian cells, including macrophages/microglia. Of interest, many of NF-*κ*B-dependent genes encode cytokines and chemokines, such as interleukin-1*β* (IL-1*β*), IL-6, IL-8, and monocyte chemoattractant protein-1, in addition to proinflammatory enzymes, such as COX-2, all of which have important roles in inflammation [47]. 

A well-defined downstream effector of ceramide is S1P, and also this sphingolipid participates in inflammatory signalling cascades [84]. Besides recruiting lymphocytes to blood and lymph, S1P may promote immune competent cell survival and proliferation but also interferes with their activation [47]. LPS activate the SK1/S1P signalling axis in several cell types including mouse macrophages [85] leading to translocation of SK1 to the plasma membrane where it converts its substrate sphingosine to S1P. It is important to note that in activated microglia SK1 expression is upregulated [59]. In general S1P elicits a wide variety of cellular responses including inflammation and can act intracellularly as a second messenger or extracellularly by binding to the G protein-coupled receptors S1P1 to S1P5 [86, 87]. The BV2 microglial cells and purified microglia from mouse primary cultures have been shown to express all or some of the five S1P receptors [59, 88]. Of interest, the reported role of S1P in cell proliferation, migration, and changes in morphology of rat astrocytes and microglia suggest a crucial role of S1P in neuroinflammatory disease conditions [89]. Moreover, it has been shown that the suppression of SK1 activity in activated mouse microglia inhibited the expression levels of TNF-*α*, IL-1*β*, and iNOS and release of TNF-*α* and NO [59]. The addition of exogenous S1P to activated cells enhanced microglia inflammatory responses, suggesting that S1P acts as an upstream factor to induce the production of proinflammatory cytokines and neurotoxic substances (such as NO). Similarly to what occurs in peripheral immune cells [90], these data suggest that the SK1/S1P pathway is involved in the inflammatory response of activated microglia in an autocrine/paracrine signalling fashion in which the secreted S1P can regulate the release of proinflammatory factors by microglia. 

Also gangliosides are capable of activating glia, thereby leading to inflammatory responses in the brain [39]. In particular, in rat brain microglia and astrocytes exposure to gangliosides can induce the production of various inflammatory mediators, such as cytokines and iNOS [39, 91–93]. This gangliosides function is mediated through the activation of different inflammation-associated signalling molecules, including NF-*κ*B, JAK, STAT (signal transducer and activator of transcription), and MAPK [39, 91, 93–95]. These results suggest that ceramide may contribute to inflammatory signalling cascades in microglia through different derivatives other than S1P.

## 5. Conclusion

The inflammatory function of microglia represents a rich field of investigation for the understanding of neuropathophysiological processes. While it is important to remember that many of neuroimmune actions of microglia are beneficial, as well as necessary for a healthy CNS, research has been particularly focussed on detrimental effects of neuroinflammation in association with CNS diseases. Defects in macrophage/microglia activity may contribute to pathogenic forms of inflammation and neurodegenerative diseases; it will thus be essential to systematically identify the mechanisms of cell modulation in different neuroinflammatory/neurodegenerative disease states and the components of the microglial cell response that contribute to disease progression. The data summarised in this review clarify that various sphingolipid systems play important roles in CNS disorders. The information discussed in this review makes us aware that much remains to be learned about sphingolipid-dependent signalling mechanisms that regulate neuroinflammation and that targeting sphingolipid pathways may prove to be a useful therapeutic strategy capable of affecting a diverse array of CNS disorders. Resolving this issue, especially in response to specific microglia activity-dependent mechanisms, will be an area of future focus that deserves attention also in therapeutic perspective.

## References

[1] Futerman AH, Hannun YA (2004). The complex life of simple sphingolipids. *EMBO Reports*.

[2] Hannun YA, Luberto C (2004). Lipid metabolism: ceramide transfer protein adds a new dimension. *Current Biology*.

[3] Payne SG, Milstien S, Spiegel S (2002). Sphingosine-1-phosphate: dual messenger functions. *FEBS Letters*.

[4] Bleicher RJ, Cabot MC (2002). Glucosylceramide synthase and apoptosis. *Biochimica et Biophysica Acta*.

[5] Yu RK, Tsai Y-T, Ariga T (2012). Functional roles of gangliosides in neurodevelopment: an overview of recent advances. *Neurochemical Research*.

[6] Hannun YA, Obeid LM (2011). Many ceramides. *Journal of Biological Chemistry*.

[7] Mandon EC, Ehses I, Rother J, van Echten G, Sandhoff K (1992). Subcellular localization and membrane topology of serine palmitoyltransferase, 3-dehydrosphinganine reductase, and sphinganine N- acyltransferase in mouse liver. *Journal of Biological Chemistry*.

[8] Ferinz K, Hurwitz R, Vielhaber G, Suzuki K, Sandhoff K (1994). Occurrence of two molecular forms of human acid sphingomyelinase. *Biochemical Journal*.

[9] Perrotta C, Bizzozero L, Cazzato D (2010). Syntaxin 4 is required for acid sphingomyelinase activity and apoptotic function. *Journal of Biological Chemistry*.

[10] Grassmé H, Cremesti A, Kolesnick R, Gulbins E (2003). Ceramide-mediated clustering is required for CD95-DISC formation. *Oncogene*.

[11] Tani M, Hannun YA (2007). Analysis of membrane topology of neutral sphingomyelinase 2. *FEBS Letters*.

[12] Perrotta C, Clementi E (2010). Biological roles of acid and neutral sphingomyelinases and their regulation by nitric oxide. *Physiology*.

[13] Kitatani K, Idkowiak-Baldys J, Hannun YA (2008). The sphingolipid salvage pathway in ceramide metabolism and signaling. *Cellular Signalling*.

[14] Nikolova-Karakashian MN, Rozenova KA (2010). Ceramide in stress response. *Advances in Experimental Medicine and Biology*.

[15] Bedia C, Levade T, Codogno P (2011). Regulation of autophagy by sphingolipids. *Anti-Cancer Agents in Medicinal Chemistry*.

[16] Spiegel S, Milstien S (2003). Sphingosine-1-phosphate: an enigmatic signalling lipid. *Nature Reviews Molecular Cell Biology*.

[17] Pyne S, Pyne NJ (2011). Translational aspects of sphingosine 1-phosphate biology. *Trends in Molecular Medicine*.

[18] Hakomori S-I (2008). Structure and function of glycosphingolipids and sphingolipids: recollections and future trends. *Biochimica et Biophysica Acta*.

[19] Maccioni HJF (2007). Glycosylation of glycolipids in the Golgi complex. *Journal of Neurochemistry*.

[20] Anderson RGW (1998). The caveolae membrane system. *Annual Review of Biochemistry*.

[21] Simons K, Ehehalt R (2002). Cholesterol, lipid rafts, and disease. *Journal of Clinical Investigation*.

[22] Hakomori S-I, Handa K, Iwabuchi K, Yamamura S, Prinetti A (1998). New insights in glycosphingolipid function: “glycosignaling domain,” a cell surface assembly of glycosphingolipids with signal transducer molecules, involved in cell adhesion coupled with signaling. *Glycobiology*.

[23] Jana A, Hogan EL, Pahan K (2009). Ceramide and neurodegeneration: susceptibility of neurons and oligodendrocytes to cell damage and death. *Journal of the Neurological Sciences*.

[24] Mencarelli C, Martinez-Martinez P (2013). Ceramide function in the brain: when a slight tilt is enough. *Cellular and Molecular Life Sciences*.

[25] Davies L, Fassbender K, Walter S (2013). Sphingolipids in neuroinflammation. *Sphingolipids in Disease*.

[26] Haughey NJ (2010). Sphingolipids in neurodegeneration. *NeuroMolecular Medicine*.

[27] Piccinini M, Scandroglio F, Prioni S (2010). Deregulated sphingolipid metabolism and membrane organization in neurodegenerative disorders. *Molecular Neurobiology*.

[28] Skaper SD, Giusti P, Facci L (2012). Microglia and mast cells: two tracks on the road to neuroinflammation. *The FASEB Journal*.

[29] van Echten-Deckert G, Walter J (2012). Sphingolipids: critical players in Alzheimer's disease. *Progress in Lipid Research*.

[30] Frick LR, Williams KL, Pittenger C (2013). Microglial dysregulation in psychiatric disease. *Clinical and Developmental Immunology*.

[31] Appel SH, Simpson EP (2001). Activated microglia: the silent executioner in neurodegenerative disease?. *Current Neurology and Neuroscience Reports*.

[32] Kingwell K (2012). Neurodegenerative disease: microglia in early disease stages. *Nature Reviews Neurology*.

[33] Perry VH, Nicoll JAR, Holmes C (2010). Microglia in neurodegenerative disease. *Nature Reviews Neurology*.

[34] Sawada M (2009). Neuroprotective and toxic changes in microglia in neurodegenerative disease. *Parkinsonism and Related Disorders*.

[35] Farooqui AA, Horrocks LA, Farooqui T (2007). Modulation of inflammation in brain: a matter of fat. *Journal of Neurochemistry*.

[36] Helmut K, Hanisch U-K, Noda M, Verkhratsky A (2011). Physiology of microglia. *Physiological Reviews*.

[37] Ransohoff RM, Perry VH (2009). Microglial physiology: unique stimuli, specialized responses. *Annual Review of Immunology*.

[38] Saijo K, Glass CK (2011). Microglial cell origin and phenotypes in health and disease. *Nature Reviews Immunology*.

[39] Jou I, Lee JH, Park SY, Yoon HJ, Joe E-H, Park EJ (2006). Gangliosides trigger inflammatory responses via TLR4 in brain glia. *American Journal of Pathology*.

[40] Ridet JL, Malhotra SK, Privat A, Gage FH (1997). Reactive astrocytes: cellular and molecular cues to biological function. *Trends in Neurosciences*.

[41] Matyszak MK (1998). Inflammation in the CNS: balance between immunological privilege and immune responses. *Progress in Neurobiology*.

[42] Zhai H, Heppner FL, Tsirka SE (2011). Microglia/macrophages promote glioma progression. *GLIA*.

[43] Schmitz G, Leuthäuser-Jaschinski K, Orsó E (2009). Are circulating monocytes as microglia orthologues appropriate biomarker targets for neuronal diseases?. *Central Nervous System Agents in Medicinal Chemistry*.

[44] Fischer H-G, Reichmann G (2001). Brain dendritic cells and macrophages/microglia in central nervous system inflammation. *Journal of Immunology*.

[45] Murray PJ, Wynn TA (2011). Protective and pathogenic functions of macrophage subsets. *Nature Reviews Immunology*.

[46] El Alwani M, Wu BX, Obeid LM, Hannun YA (2006). Bioactive sphingolipids in the modulation of the inflammatory response. *Pharmacology and Therapeutics*.

[47] Nixon GF (2009). Sphingolipids in inflammation: pathological implications and potential therapeutic targets. *British Journal of Pharmacology*.

[48] Singh I, Pahan K, Khan M, Singh AK (1998). Cytokine-mediated induction of ceramide production is redox-sensitive: implications to proinflammatory cytokine-mediated apoptosis in demyelinating diseases. *Journal of Biological Chemistry*.

[49] Lee J-T, Xu J, Lee J-M (2004). Amyloid-*β* peptide induces oligodendrocyte death by activating the neutral sphingomyelinase-ceramide pathway. *Journal of Cell Biology*.

[50] Pahan K, Sheikh FG, Khan M, Namboodiri AMS, Singh I (1998). Sphingomyelinase and ceramide stimulate the expression of inducible nitric-oxide synthase in rat primary astrocytes. *Journal of Biological Chemistry*.

[51] Won J-S, Im Y-B, Khan M, Singh AK, Singh I (2004). The role of neutral sphingomyelinase produced ceramide in lipopolysaccharide-mediated expression of inducible nitric oxide synthase. *Journal of Neurochemistry*.

[52] Hannun YA, Obeid LM (2008). Principles of bioactive lipid signalling: lessons from sphingolipids. *Nature Reviews Molecular Cell Biology*.

[53] Lièvremont J-P, Sciorati C, Morandi E (1999). The p75(NTR)-induced apoptotic program develops through a ceramide- caspase pathway negatively regulated by nitric oxide. *Journal of Biological Chemistry*.

[54] Kosicek M, Hecimovic S (2013). Phospholipids and Alzheimer's disease: alterations, mechanisms and potential biomarkers. *International Journal of Molecular Sciences*.

[55] Cutler RG, Kelly J, Storie K (2004). Involvement of oxidative stress-induced abnormalities in ceramide and cholesterol metabolism in brain aging and Alzheimer’s disease. *Proceedings of the National Academy of Sciences of the United States of America*.

[56] Wenk GL (2003). Neuropathologic changes in Alzheimer’s disease. *Journal of Clinical Psychiatry*.

[57] Puglielli L, Ellis BC, Saunders AJ, Kovacs DM (2003). Ceramide stabilizes *β*-site amyloid precursor protein-cleaving enzyme 1 and promotes amyloid *β*-peptide biogenesis. *Journal of Biological Chemistry*.

[58] Dheen ST, Kaur C, Ling E-A (2007). Microglial activation and its implications in the brain diseases. *Current Medicinal Chemistry*.

[59] Nayak D, Huo Y, Kwang WXT (2010). Sphingosine kinase 1 regulates the expression of proinflammatory cytokines and nitric oxide in activated microglia. *Neuroscience*.

[60] Tamboli IY, Hampel H, Tien NT (2011). Sphingolipid storage affects autophagic metabolism of the amyloid precursor protein and promotes A*β* generation. *Journal of Neuroscience*.

[61] Won J-S, Singh AK, Singh I (2007). Lactosylceramide: a lipid second messenger in neuroinflammatory disease. *Journal of Neurochemistry*.

[62] Jeyakumar M, Smith DA, Williams IM (2004). NSAIDs increase survival in the Sandhoff disease mouse: synergy with N-butyldeoxynojirimycin. *Annals of Neurology*.

[63] Jeyakumar M, Thomas R, Elliot-Smith E (2003). Central nervous system inflammation is a hallmark of pathogenesis in mouse models of GM1 and GM2 gangliosidosis. *Brain*.

[64] Crawley AC, Walkley SU (2007). Developmental analysis of CNS pathology in the lysosomal storage disease *α*-mannosidosis. *Journal of Neuropathology and Experimental Neurology*.

[65] Desplats PA, Denny CA, Kass KE (2007). Glycolipid and ganglioside metabolism imbalances in Huntington’s disease. *Neurobiology of Disease*.

[66] Denny CA, Desplats PA, Thomas EA, Seyfried TN (2010). Cerebellar lipid differences between R6/1 transgenic mice and humans with Huntington’s disease. *Journal of Neurochemistry*.

[67] Dunbar GL, Sandstrom MI, Rossignol J, Lescaudron L (2006). Neurotrophic enhancers as therapy for behavioral deficits in rodent models of Huntington’s disease: use of gangliosides, substituted pyrimidines, and mesenchymal stem cells. *Behavioral and Cognitive Neuroscience Reviews*.

[68] Maglione V, Marchi P, Di Pardo A (2010). Impaired ganglioside metabolism in Huntington’s disease and neuroprotective role of GM1. *Journal of Neuroscience*.

[69] Di Pardo A, Maglione V, Alpaugh M (2012). Ganglioside GM1 induces phosphorylation of mutant huntingtin and restores normal motor behavior in Huntington disease mice. *Proceedings of the National Academy of Sciences of the United States of America*.

[70] Snider AJ, Orr Gandy KA, Obeid LM (2010). Sphingosine kinase: role in regulation of bioactive sphingolipid mediators in inflammation. *Biochimie*.

[71] Göggell R, Winoto-Morbach S, Vielhaber G (2004). PAF-mediated pulmonary edema: a new role for acid sphingomyelinase and ceramide. *Nature Medicine*.

[72] Masini E, Giannini L, Nistri S (2008). Ceramide: a key signaling molecule in a guinea pig model of allergic asthmatic response and airway inflammation. *Journal of Pharmacology and Experimental Therapeutics*.

[73] Sakata A, Ochiai T, Shimeno H (2007). Acid sphingomyelinase inhibition suppresses lipopolysaccharide-mediated release of inflammatory cytokines from macrophages and protects against disease pathology in dextran sulphate sodium-induced colitis in mice. *Immunology*.

[74] Jung JS, Shin KO, Lee YM (2013). Anti-inflammatory mechanism of exogenous C2 ceramide in lipopolysaccharide-stimulated microglia. *Biochimica et Biophysica Acta*.

[75] Teichgräber V, Ulrich M, Endlich N (2008). Ceramide accumulation mediates inflammation, cell death and infection susceptibility in cystic fibrosis. *Nature Medicine*.

[76] Becker KA, Riethmüller J, Zhang Y, Gulbins E (2010). The role of sphingolipids and ceramide in pulmonary inflammation in cystic fibrosis. *Open Respiratory Medicine Journal*.

[77] Sun Y, Fox T, Adhikary G, Kester M, Pearlman E (2008). Inhibition of corneal inflammation by liposomal delivery of short-chain, C-6 ceramide. *Journal of Leukocyte Biology*.

[78] Józefowski S, Czerkies M, Łukasik A (2010). Ceramide and ceramide 1-phosphate are negative regulators of TNF-*α* production induced by lipopolysaccharide. *Journal of Immunology*.

[79] Rozenova KA, Deevska GM, Karakashian AA, Nikolova-Karakashian MN (2010). Studies on the role of acid sphingomyelinase and ceramide in the regulation of tumor necrosis factor *α* (TNF*α*)-converting enzyme activity and TNF*α* secretion in macrophages. *Journal of Biological Chemistry*.

[80] Hsu Y-W, Chi K-H, Huang W-C, Lin W-W (2001). Ceramide inhibits lipopolysaccharide-mediated nitric oxide synthase and cyclooxygenase-2 induction in macrophages: effects on protein kinases and transcription factors. *Journal of Immunology*.

[81] Cho YH, Lee CH, Kim SG (2003). Potentiation of lipopolysaccharide-inducible cyclooxygenase 2 expression by C2-ceramide via c-Jun N-terminal kinase-mediated activation of CCAAT/enhancer binding protein *β* in macrophages. *Molecular Pharmacology*.

[82] Haimovitz-Friedman A, Cordon-Cardo C, Bayoumy S (1997). Lipopolysaccharide induces disseminated endothelial apoptosis requiring ceramide generation. *Journal of Experimental Medicine*.

[83] Schutze S, Potthoff K, Machleidt T, Berkovic D, Wiegmann K, Kronke M (1992). TNF activates NF-*κ*B by phosphatidylcholine-specific phospholipase C- induced “acidic” sphingomyelin breakdown. *Cell*.

[84] Weigert A, Weis N, Brüne B (2009). Regulation of macrophage function by sphingosine-1-phosphate. *Immunobiology*.

[85] Hammad SM, Crellin HG, Wu BX, Melton J, Anelli V, Obeid LM (2008). Dual and distinct roles for sphingosine kinase 1 and sphingosine 1 phosphate in the response to inflammatory stimuli in RAW macrophages. *Prostaglandins and Other Lipid Mediators*.

[86] Ozaki H, Hla T, Lee M-J (2003). Sphingosine-1-phosphate signaling in endothelial activation. *Journal of Atherosclerosis and Thrombosis*.

[87] Rosen H, Goetzl EJ (2005). Sphingosine 1-phosphate and its receptors: an autocrine and paracrine network. *Nature Reviews Immunology*.

[88] Tham C-S, Lin F-F, Rao TS, Yu N, Webb M (2003). Microglial activation state and lysophospholipid acid receptor expression. *International Journal of Developmental Neuroscience*.

[89] Bassi R, Anelli V, Giussani P, Tettamanti G, Viani P, Riboni L (2006). Sphingosine-1-phosphate is released by cerebellar astrocytes in response to bFGF and induces astrocyte proliferation through Gi-protein-coupled receptors. *GLIA*.

[90] Melendez AJ (2008). Sphingosine kinase signalling in immune cells: potential as novel therapeutic targets. *Biochimica et Biophysica Acta*.

[91] Kim OS, Park EJ, Joe E-H, Jou I (2002). JAK-STAT signaling mediates gangliosides-induced inflammatory responses in brain microglial cells. *Journal of Biological Chemistry*.

[92] Kanda N, Watanabe S (2001). Gangliosides GD1b, GT1b, and GQ1b enhance IL-2 and IFN-*γ* production and suppress IL-4 and IL-5 production in phytohemagglutinin-stimulated human T cells. *Journal of Immunology*.

[93] Ryu JK, Shin WH, Kim J (2002). Trisialoganglioside GT1b induces in vivo degeneration of nigral dopaminergic neurons: role of microglia. *GLIA*.

[94] McLaurin J, Franklin T, Fraser PE, Chakrabartty A (1998). Structural transitions associated with the interaction of Alzheimer *β*- amyloid peptides with gangliosides. *Journal of Biological Chemistry*.

[95] Yang M-S, Park EJ, Sohn S (2002). Interleukin-13 and -4 induce death of activated microglia. *GLIA*.

